# Mediterranean Diet and Phase Angle in a Sample of Adult Population: Results of a Pilot Study

**DOI:** 10.3390/nu9020151

**Published:** 2017-02-17

**Authors:** Luigi Barrea, Giovanna Muscogiuri, Paolo Emidio Macchia, Carolina Di Somma, Andrea Falco, Maria Cristina Savanelli, Annamaria Colao, Silvia Savastano

**Affiliations:** 1 I.O.S. COLEMAN Srl, Acerra, 80011 Naples, Italy; giovanna.muscogiuri@gmail.com (G.M.); falco.and@gmail.com (A.F.); cristysav@hotmail.com (M.C.S.); 2 Dipartimento di Medicina Clinica e Chirurgia, Unit of Endocrinology, Federico II University Medical School of Naples, Via Sergio Pansini 5, 80131 Naples, Italy; pmacchia@unina.it (P.E.M.); colao@unina.it (A.C.); sisavast@unina.it (S.S.); 3 IRCCS SDN, Napoli Via Gianturco 113, 80143 Naples, Italy; cdisomma@unina.it

**Keywords:** mediterranean diet, phase angle, bioelectrical impedance analysis, PREDIMED score

## Abstract

The Mediterranean diet is a healthy dietary pattern known to actively modulate the cell membrane properties. Phase angle (PhA) is a direct measure by Bioelectrical Impedance Analysis (BIA) used as marker of cell membrane integrity. Both food behaviour and PhA are influenced by age, sex and body weight. The aim of this study was to cross-sectionally evaluate the association between the adherence to Mediterranean diet and PhA in 1013 healthy adult patients stratified according to sex, age, and body mass index (BMI). The adherence to the Mediterranean diet was evaluated using the PREvención con DIeta MEDiterránea (PREDIMED) questionnaire. PhA was calculated by BIA phase-sensitive system (50 kHz BIA 101 RJL, Akern Bioresearch, Florence, Italy Akern). In both sexes, at ROC analysis a PREDIMED score ≥ 6 predicted a PhA beyond the median value. At the multivariate analysis, among PREDIMED score, age, and BMI, the PREDIMED score was the major determinant of PhA, explaining 44.5% and 47.3% of PhA variability, in males and females respectively (*p* < 0.001). A novel association was reported between the adherence to the Mediterranean diet and PhA, independently of sex, age, and body weight. This association uncovered a new potential benefit of the Mediterranean diet on health outcomes, as in both sexes higher adherence to the Mediterranean diet was associated to larger PhAs, as expression of cell membrane integrity.

## 1. Introduction

Diet is one of the most important environmental factors. In particular, the Mediterranean diet is considered a healthy dietary pattern, nutritionally adequate and complete and easy to follow [1].

The Mediterranean diet is traditionally based on some common dietary characteristics and lifestyle behaviours of the Mediterranean countries and, to date, is the most studied at the level of evidence-based medicine [2]. In particular, studies evaluating the effects of basic components of the Mediterranean diet on the physicochemical properties of membrane lipoproteins raise the possibility that Mediterranean diet can actively modulate cell membrane properties [3,4]. However, it is well known that gender, age and obesity influence food choice behaviours [5]. In particular, women’s dietary profiles is characterized by a higher carbohydrates intake, including fruit and vegetables [6], while young men show a low adherence to the Mediterranean diet [7].

Phase angle (PhA), a direct measure by Bioelectrical Impedance Analysis (BIA), is used as a marker of cell membrane integrity and body cell mass [8,9] and a predictor of morbidity and mortality in various diseases [10,11]. PhA is calculated as the relationship between the resistance (R) of tissues, which is mainly dependent on tissue hydration, and the reactance (Xc) of tissues associated with cellularity, cell size and integrity of the cell membrane [12,13,14]. Lower PhAs appear to be consistent with either cell death or a breakdown in the selective permeability of the cell membrane, while larger PhAs are associated with large quantities of intact, healthy cell membranes and body cell mass [15]. In healthy population, sex, age, and body mass index (BMI) are the major determinants of PhA [9,16]. In particular, PhA is known to be higher in healthy male subjects in comparison to female subjects due to lower fat mass and higher muscle mass percentage in the formers. On the other hand, age and body weight variably affect the amount of fat mass, muscle cells, and the functional status of cell membranes. Thus, there is a biphasic correlation of PhA with age and BMI, with positive correlations in younger and normal weight/overweight subjects, and an inverse correlations in older age and obesity classes [13,17]. In addition, there is a significant difference in PhA between healthy and disease states [16]. In disease conditions, including malnutrition, PhA is frequently lower than normal and its use has been recommended as a prognostic marker of mortality in various chronic diseases [11], including cancer, and is associated with risk of morbidity in diabetes [18], obesity [19], and psoriasis [20,21]. On the other hand, the PhA increases with improving clinical status [15,22]. 

An association between PhA and nutritional status has been reported in free-living individuals. In particular, in a cross-sectional study recently published by de França NAG et al. [23], consumed portions of single food components, such as extra-virgin olive oil, cereals, legumes, and meat, showed a weak, but significant positive correlation with PhA [23]. However, diet is a complex combination of foods from various groups and nutrients, and some nutrients are highly correlated. Thus, it is challenging, to separate the effect of a single nutrient or food group from others in free-living populations [24]. The possible influence a healthful eating pattern, such as Mediterranean diet, on PhA values has been never investigated previously and, as long as we know, data on the association between Mediterranean diet and PhA on adult sample population of both sexes are still lacking.

Taking into account the influence of sex, age, and BMI on both the Mediterranean eating pattern and the PhA, the aim of this observational study was to evaluate the association between the adherence to the Mediterranean diet and PhA values in a sample of adult population, stratified according to categories of sex, age, and BMI.

## 2. Subjects and Methods

### 2.1. Design and Setting

This is a cross-sectional observational study carried out at the Campus Salute, a project to investigate the role of lifestyle in preventing chronic diseases supported by the Campus Salute association of Campania [25], and at the Department of Clinical Medicine and Surgery, Unit of Endocrinology, University Federico II, Naples (Italy), from October 2011 to March 2016. The work has been carried out in accordance with the Code of Ethics of the World Medical Association (Declaration of Helsinki) for experiments involving humans, and it has been approved by the Ethical Committee of the University of Naples “Federico II” Medical School (n. 239/11). The purpose of the protocol was explained to all the study participants, and written informed consent was obtained. This cross-sectional observational study was registered at clinicaltrials.gov (NCT02840968).

### 2.2. Population Study

Recruitment strategies included attendance at the outpatient obesity clinics at our Department or the outdoor hospital of the Campus Salute. The study is part of a large database started in 2010 to investigate the health status of the general population of Campania Region as analysed by the free consultation, visit, and diagnostics for people coming to the outdoor hospital held in different public squares of our Region. A sample of 1464 of adult subjects of both sexes aged 18–59 years was recruited. All subjects were Caucasians and healthy (defined as absence of a clinical condition that potentially influences fluid balance, i.e., renal, endocrine, or myocardial disease, ascertained by participant questionnaire). All female subjects were non-pregnant and non-lactating, and were evaluated in the follicular phase of the menstrual cycle. A full medical history, including drug use, was collected. 

**Figure 1:**
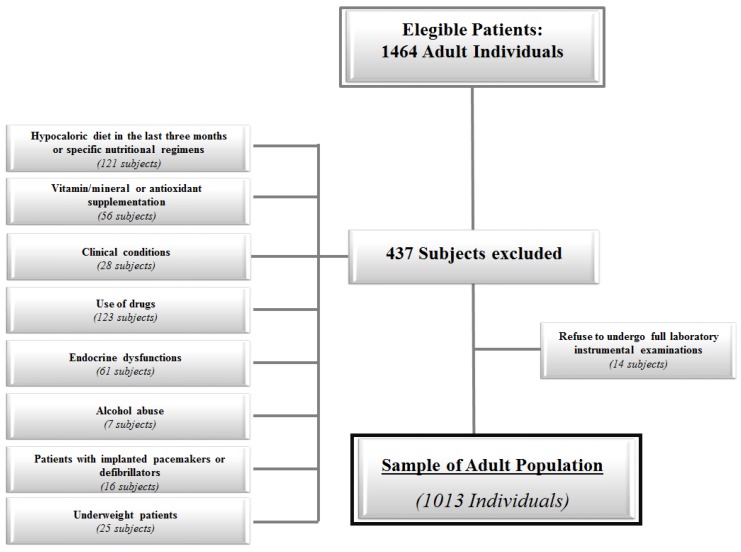
Flow chart of study design.

Criteria for exclusion from the study were: (a) hypocaloric diet in the last three months or specific nutritional regimens, including vegan or vegetarian diets (121 and four subjects, respectively); (b) vitamin/mineral or antioxidant supplementation (56 subjects); (c) clinical conditions that could influence fluid balance, including liver or renal failure, cancer, and acute or chronic inflammatory diseases, based on a complete medical examination and laboratory investigations (28 subjects); (d) occasional or current of use of drugs that could influence fluid balance, including non-steroidal anti-inflammatory drugs (23 subjects), hormone replacement therapy (54 subjects), diuretics (22 subjects), weight-loss medications (four subjects), anticonvulsants and psychotropic agents (14 subjects), laxative use (six subjects); (e) altered thyroid hormone function tests or thyroid hormone treatment (61 subjects); (f) alcohol abuse according to the Diagnostic and Statistical Manual of Mental Disorders (DSM)-V diagnostic criteria (seven subjects); (g) patients with implanted pacemakers or defibrillators because of the theoretical possibility of interference with the device activity due to the field of current induced by the impedance measurements (16 subjects); (h) underweight patients with BMI < 18.5 kg/m^2^ (22 females and 3 males). Finally, 14 subjects dropped out from the study since they refused to participate. The flow chart of study subjects is shown in Figure 1. Measurements were performed between 8 a.m. and 12 p.m. All subjects were measured after an overnight fast.

### 2.3. Anthropometric Measurements 

The measurements were made in a standard way by one operator (a nutritionist experienced in providing nutritional assessment and body composition). All anthropometric measurements were taken with subjects wearing only light clothes and without shoes. In each subject, weight and height were measured to calculate the BMI (weight (kg) divided by height squared (m^2^), kg/m^2^). Height was measured to the nearest 0.5 cm using a wall-mounted stadiometer (Seca 711; Seca, Hamburg, Germany). Body weight was determined to the nearest 0.1 kg using a calibrated balance beam scale (Seca 711; Seca, Hamburg, Germany). BMI was classified according to WHO’s criteria with normal weight: 18.5–24.9 kg/m^2^; overweight, 25.0–29.9 kg/m^2^; grade I obesity, 30.0–34.9 kg/m^2^; grade II obesity, 35.0–39.9 kg/m^2^; grade III obesity ≥40.0 kg/m^2^.

### 2.4. Adherence to the Mediterranean Diet

The adherence to the Mediterranean diet was evaluated using the previously validated 14-item questionnaire for the assessment of PREvención con DIeta MEDiterránea (PREDIMED) [26]. A qualified Nutritionist administered the questionnaire during a face-to-face interview to all the enrolled subjects. Briefly, for each items was assigned score 1 and 0; PREDIMED score was calculated as follows: 0–5, lowest adherence; score 6–9, average adherence; score ≥ 10, highest adherence [26].

### 2.5. Bioelectrical Impedance Analysis

Bioelectrical impedance analysis was performed using a BIA phase-sensitive system by experienced observers (an 800-µA current at a frequency single-frequency of 50 kHz BIA 101 RJL, Akern Bioresearch, Florence, Italy) [27]. Based on the European Society of Parenteral and Enteral Nutrition (ESPEN) guidelines [28], all participants were supine with limbs slightly spread apart from the body, refrained from eating, drinking, and exercising for six hours and no alcohol within 24 h before testing. Shoes and socks were removed and contact areas were scrubbed with alcohol immediately before electrode placement. Electrodes (BIATRODES Akern Srl; Florence, Italy) were placed proximal to the phalangeal–metacarpal joint on the dorsal surface of the right hand and distal to the transverse arch on the superior surface of the right foot. Sensor electrodes were placed at the midpoint between the distal prominence of the radius and ulna of the right wrist, and between the medial and lateral malleoli of the right ankle. See Kushner et al. [29] for a detailed description of the measurement procedure. All measurements were performed under strictly standardized conditions by the author, using the same device in order to avoid interobserver and interdevice variability. The instrument was routinely checked with resistors and capacitors of known values. Reliability for within-day and between-day measurements by the same observer were <2% for R, <2.5% for Xc, and <3.3% for R, <2.8% for Xc, respectively. The coefficient of variation (CV) of repeated measurements of R and Xc at 50 kHz was assessed in 10 patients (5 males and 5 females) by the same observer: CVs were 1.8% for R and 1.6% for Xc. The PhA was derived from conditions under 50 kHz according to the following formula: PhA (°, degrees) = arctangent Xc/R ((Xc/R) × (180/π)).

### 2.6. Statistical Analysis

Results are expressed as mean ± SD or as median plus range, according to variable distributions evaluated by Kolmogorov-Smirnov test (*p* < 0.01), 5th and 10th percentile. The chi square (χ^2^) test was used to determine the significance of differences in frequency distribution. For tertile of PhA, continuous variables were compared by analysis of variance (ANOVA) when the distributions were normal and the variances were equivalent; otherwise, they were compared by using the Kruskal-Wallis test, followed by Bonferroni test as post-hoc test. Differences in PREDIMED score and PhA between male and female participants were analyzed by unpaired *t* test, and among the age and BMI groups were analyzed by ANOVA test, with the Bonferroni test as post-hoc test. The correlations between study variables were performed using Pearson r or Spearman’s rho correlation coefficients according to the variable’s distribution.

Receiver operator characteristic (ROC) curve analysis was performed to determine sensitivity and specificity, area under the curve (AUC), and confidence intervals (CI), as well as cut-off values for PREDIMED score in detecting PhA above the median values in male and female participants. Test AUC for ROC analysis was also performed. We want show that AUC resulted 0.957 for a particular test is significant from the null hypothesis value 0.5 (meaning no discriminating power), than we enter 0.957 for AUC ROC and 0.5 for null hypothesis values. For α level we selected 0.05 type I error and for β level we selected 0.20 type II error. To analyze the discriminant value of the PREDIMED score, the PhA AUCs were compared using a linear logistic regression model including sex, age, and BMI as independent variables, before and after the addition of PREDIMED score in the model. Two multiple regression analysis models (stepwise method), expressed as Beta (β), *t*, *R*^2^, and 95% confidence interval (CI), with PhA as dependent variable were used to estimate the predictive values of PREDIMED score, BMI and age in both male and female participants. In these analyses, we entered only those variables that had a *p*-value < 0.05 in the univariate analysis (partial correlation). To avoid multicollinearity, variables with a variance inflation factor (VIP) > 10 were excluded. Values ≤ 5% were considered statistically significant. Data were stored and analyzed using the MedCalc^®^ package (Version 12.3.0 1993–2012 MedCalc Software bvba—MedCalc Software, Mariakerke, Belgium). Proportional odds model was carried out using the R Project for Statistical Computing 2014 [30].

## 3. Results

The final study population consisted of 1013 subjects (461 males; 45.5%). The adult population sample was largely representative of the adult population living in the Campania Region with regard to sex (χ^2^ = 0.08, *p* = 0.777) [31]. The descriptive characteristics of the study population were given in Table 1. According to PREDIMED score, the majority of subjects included in this study reached a high-average adherence to the Mediterranean diet, while only one third of the subjects reported a low adherence. Significantly larger PhA values were found in males than in females (*p* < 0.001). The study participants were divided into the tertile of PhA, according to sex. The data are presented in two parts of the supplementary materials tables (Table S1). Part 1 shows the tertile of the PhA in males and part 2 shows the tertile of the PhA in females. In both males and females, subjects in the lowest tertile of PhA values were older, had a higher BMI and showed the lower adherence to the Mediterranean diet.

The study population was divided according to sex, age, and BMI classes as follows. In particular, in the study population 5 BMI categories (normal weight, overweight, and obesity grade I–III) were stratified into 4 age groups (18–28, 29–38, 39–48, and 49–58 years). In Table 2, means (±SD) and the respective 10th and 5th percentiles for PREDIMED score were shown by sex, age, and BMI categories. In particular, across all BMI categories, females showed an overall higher adherence to Mediterranean diet compared with males up to 18–28 age years. In addition, within each sex and BMI group, we observed an overall significant increase of the PREDIMED score up to 29–38 years, with a subsequent decline along with increasing age. In contrast, within each sex and age group PREDIMED score tended to decrease with increasing BMI. A similar trend was evidenced for means (±SD) and the respective 10th and 5th percentiles for PhA across sex, age, and BMI categories, as shown in Table 3. As expected, across all BMI and age categories PhA was significantly greater in males than in females. In addition, we observed that within each sex and BMI group, PhA tended to increase with increasing age up to 29–38 years, except for the normal weight men whose PhA continued to increase up to 39–48 years. Thereafter, as expected, in both sexes in each BMI categories, PhA decreased with increasing age. In particular, within each age group PhA tended to increase with increasing BMI up to a value of 25–30 kg/m^2^ in both females and males, while decreasing in higher BMI groups. Means (±SD) of PhA for each 14-PREDIMED score were reported in Table 4. In both sexes, the largest PhA values were observed among the subjects reaching the highest PREDIMED score.

**Figure 2:**
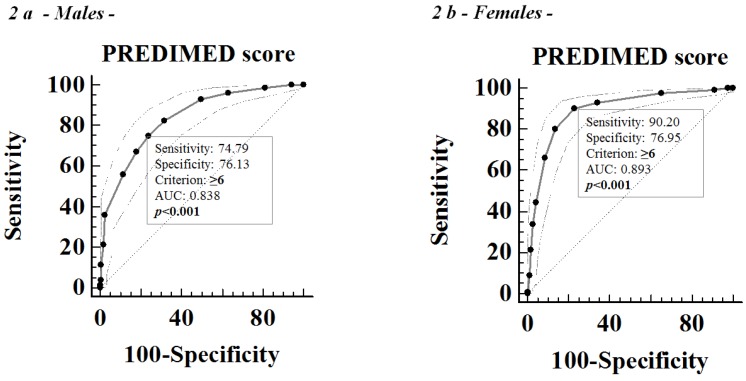
Receiver operating characteristic analysis (ROC) for predictive values of PREDIMED score in detecting PhA values above the median values. The most sensitive and specific cut-off of the PREDIMED score to predict PhA above the median value (6.2° and 5.6° in male and female participants, respectively), was ≥6 in males participants (**a**) and females participants (**b**). PREDIMED, PREvención con DIeta MEDiterránea; PhA, Phase Angle, AUC, Area Under Curve.

**Figure 3:**
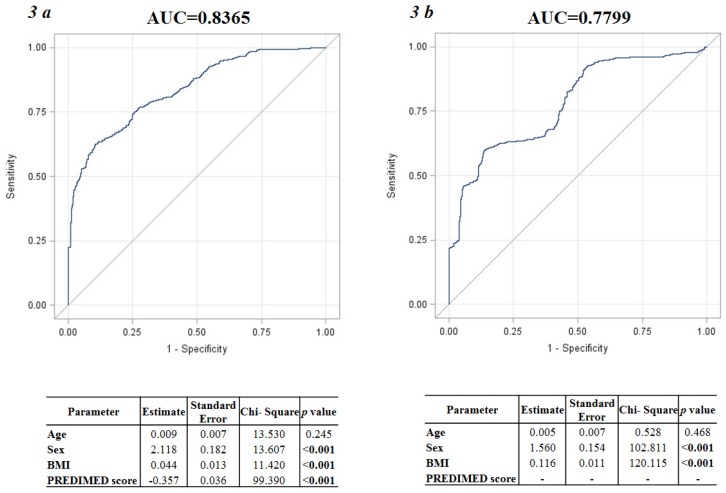
Linear logistic regression analysis using the PhA AUC as dependent variable and sex, age, and BMI as independent variables, before and after the addition of PREDIMED score in the model. At the fitting model, the AUC significantly increased by 7% once the PREDIMED score was added to the baseline model (AUC = 0.8365 vs. AUC = 0.7799; (**a**,**b**) respectively). *p* value in bold type denotes a significant difference (*p* < 0.05). PhA, Phase Angle; AUC, Area Under Curve; PREDIMED, PREvención con DIeta MEDiterránea; BMI, Body Mass Index.

ROC analysis for predictive values of the adherence of Mediterranean diet in detecting the PhA above the median value, according to sex, was reported in Figure 2a,b. The number of cases required for each group was set at 23 and 17, from the AUC 0.81 and 0.86, for males and females, respectively. Based on ROC curves, the most sensitive and specific cut-off of the PREDIMED score to predict PhA above the median value (6.2° and 5.6° in male and female participants, respectively) was ≥6 in both sexes. In addition, at the linear logistic regression analysis of the PhA AUCs obtained including sex, age, and BMI as independent variables, before and after the addition of PREDIMED score in the model, the AUC significantly increased by 7% once the PREDIMED score was added to the baseline model (AUC = 0.8365 vs. AUC = 0.7799; Figure 3a,b).

### Correlation Studies

**Figure 4:**
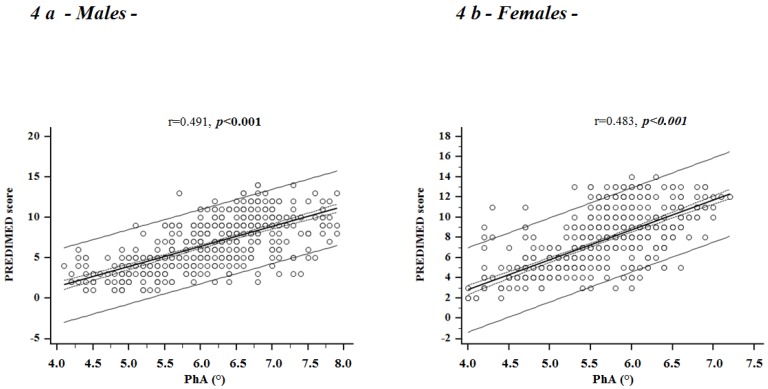
Correlations between PREDIMED score and PhA in male (**a**) and female participants (**b**), adjusted for age and BMI. A significant positive correlation was observed between PREDIMED score and PhA in both sexes, independently of age and BMI. PREDIMED, PREvención con DIeta MEDiterránea; PhA, Phase Angle.

In Figure 4 we reported the correlations between the adherence to the Mediterranean diet and PhA in male (a) and female (b) participants, adjusted for age and BMI, while in Table 5 we showed the separate analyses of correlations stratified according to sex and BMI categories. A significant positive correlation was observed between PREDIMED score and PhA in both sexes along BMI categories in unadjusted data. Of interest, PhA remained positively correlated with PREDIMED score independently of age and BMI. 

The association between the adherence to Mediterranean diet and BMI and between PhA and BMI were evaluated and provided as Tables S2 and S3, respectively. A significant positive correlation was observed between PREDIMED score and BMI in normal weight in both sexes in unadjusted data. Along with increasing BMI, this correlation became negative and remained significant also after adjusting for age, in each sex and BMI categories (Table S2). Contrarily, the association between PhA and BMI showed a gender dimorphism, as in male participants there was a positive association between PhA and BMI in normal weight and overweight categories, whereas in females this positive association was evident in only in normal weight category. In both sexes, along with increasing BMI, a negative association was present starting from obesity III category. This association remained significant also after adjusting for age (Table S3). 

To compare the relative predictive power of the variables found to be significantly associated with PhA, we performed two stepwise multiple linear regression analysis models that included PREDIMED score as measures of adherence of the Mediterranean diet, age and BMI, separately in male and female participants. Using these models, PREDIMED score entered at the first step and appeared to be the factor exerting the most powerful influence on PhA, explaining 44.5% and 47.3% of PhA variability, in male and female respectively. BMI entered at the second step, followed by age, in males and females, respectively. The total proportion of PhA variability explained by these models were 52.1% and 50.8% in males and females, respectively (Table 6).

## 4. Discussion

In this cross-sectional observational study we evaluated the association between the adherence to the Mediterranean diet and PhA in a sample of adult population. Taking into account that both the Mediterranean eating pattern and the PhA are influenced by sex, age, and BMI, we stratified the study participants according to these variables. A novel association was reported between the adherence to the Mediterranean diet, a dietary pattern associated with positive effects on health and well-being [1] and PhA, a direct measure by BIA used as a marker of cell membrane integrity and predictor of morbidity and mortality in various diseases [8,9,10,11,12]. Because there is a great body of evidence that the Mediterranean diet is a healthy dietary pattern, the association between higher PREDIMED score and PhA is not surprising. Actually, of interest our data showed that this association was independently of sex, age, and body weight. In fact, we found that in both sexes, subjects in the lowest tertile of PhA values were older, had a higher BMI and showed the lower adherence to the Mediterranean diet, and the largest PhAs were associated with the highest adherence to the Mediterranean diet across all age and BMI categories. In addition, we reported the corresponding mean value of PhA for each score of adherence to the Mediterranean diet. Based on ROC curve analysis, the most sensitive and specific cut-offs for the score of adherence to the Mediterranean diet to predict PhAs larger than median values were ≥6 in both male and female participants. Furthermore, the PREDIMED score has 7% additional value for predicting PhA beyond the predictive discrimination of the factors that commonly affect PhA, such as sex, age, and BMI. At the multivariate analysis, among variables related with PhA, including age, body weight and the score of adherence to the Mediterranean diet, the latter was the major determinant of PhA in both sexes. 

Although it was not a primary aim of this study, a clear gender difference was observed in the adherence to the Mediterranean diet in our study population, as female participants showed an overall higher adherence to the Mediterranean diet compared with male counterpart. However, this gender difference was significant in only young categories across all BMI classes. In both sexes, the adherence to the Mediterranean diet was higher in the median age categories in each BMI class, and in each age categories in normal weight compared with higher BMI classes. Additionally, in line with observational and prospective studies in different population settings, our findings showed that the adherence to the Mediterranean diet and BMI were significantly negatively correlated [26,32,33]. In particular, after adjusting for age, the adherence to the Mediterranean diet decreased across the higher BMI classes in both sexes, indicating a stronger correlation with BMI than with age. However, we found that this correlation showed a biphasic trend characterized by a positive correlation in normal weight categories, and a negative correlation in the other BMI categories in both sexes. 

Large epidemiological studies have clearly demonstrated that PhA varies differently across sex, age or BMI categories [13,14]. Our data consistently confirmed a clear sexual dimorphism in PhAs across higher BMI classes, as larger PhAs were found among male in comparison to female participants, as expression of the higher quantities of intact cell membranes and lean body mass in the formers. Similarly, larger PhAs were found among younger individuals in both sexes. As regard BMI, it is well-known that PhA is positively related to increasing BMI, due the increased amount of cell membranes in both fat and muscle cells [14]. This positive relationship is inverted at higher BMI values, likely related to the more prevalent accrual of fat mass rather than lean body mass along with increasing BMI. Accordingly, we found that, in both sexes, larger PhAs were found up to the normal weight categories, with a subsequent decrease in the highest BMI classes. However, in overweight categories the relationship between PhA and BMI is different between male and female participants, as the positive correlation is maintained only in the former, while it disappeared in the females, likely due to the effects of the gender-specific greater accrual of fat mass among females and lean mass among males along with increasing BMI. Of interest, besides the well-known association of PhA with sex, age and BMI, we found a novel association between the adherence to the Mediterranean diet and PhA, showing also that this relationship was largely independent of sex, age, and BMI, and that the PREDIMED score was the major predictor of PhA. In addition, we provided the mean value of PhA for each 14-PREDIMED score. Considering that expert Nutritionists are required for the assessment, execution and especially for interpretation of BIA measurements, in particular PhA, the determination of a specific cut-off value for the score of adherence to Mediterranean diet on larger population samples might help identifying high-risk subjects with small PhAs who could get benefit of careful dietary interventions in clinical setting.

Limitations to this study warrant some considerations. The cross-sectional nature of this study did not allow to identify any causal association between Mediterranean diet and PhA and to clearly determine the prognostic value of the adherence to the Mediterranean diet for predicting the PhA. Moreover, the suggested cut-off value of the PREDIMED score for identify the large PhAs in our present study should be viewed with caution until results of studies in larger patient populations have become available to perform an appropriate cross-validation. A further limitation might concern the misclassification error for food consumption using questionnaires, such as the 14-item PREMIMED brief questionnaire. Nevertheless, this less time-demanding and less expensive questionnaire, that requires less collaboration from participants than the usual full length food frequency questionnaires or other more comprehensive methods, has previously been validated by different clinical observational and interventional studies and accurately classified participants with respect to scoring for different validated methods [26]. In addition, the strength of this study includes the stratification of PhA values for sex, age and BMI-specific categories, making possible the comparisons across subjects independently of the age and BMI structure of the studied samples, even when the age and BMI of the population vary widely. 

In conclusion, we reported a novel positive association between the Mediterranean diet and the PhA independent of sex, age and BMI. This association might uncover a further potential mechanism for the Mediterranean diet relating this healthy pattern of nutrient intake to health outcomes through the PhA in both sexes. In this, the results of this study would recommend the nutrition assessment as good clinical practice in the evaluation of PhA in clinical settings. Future large dietary intervention trials will be critical for elucidate the beneficial effects of the Mediterranean diet on the PhA.

## Figures and Tables

**Table 1:** Anthropometric measures, adherence to the Mediterranean diet and bioelectrical variables.

Parameters	Subjects *n* = 1013
Age (years)	37 (18–58)
Anthropometric measures	
Weight (kg)	95.0 (47.0–186.0)
Height (m)	1.70 (1.50–1.92)
BMI (kg/m^2^)	33.5 (19.5–57.9)
Normal weight *n* (%)	164 (16.2%)
Overweight *n* (%)	202 (19.9%)
Obesity grade I *n* (%)	195 (19.2%)
Obesity grade II *n* (%)	203 (20.0%)
Obesity grade III *n* (%)	249 (24.6%)
Adherence to the Mediterranean diet	
PREDIMED score	7.1 ± 3.0
Low adherence *n* (%)	382 (37.7%)
Average adherence *n* (%)	398 (39.3%)
High adherence *n* (%)	233 (23.0%)
Bioelectrical variables	
R (Ohm, Ω)	471.0 (250.0–781.0)
Xc (Ohm, Ω)	48.0 (20.0–85.0)
PhA (°)	5.8 ± 0.8
PhA (°) Male	6.1 ± 0.8
PhA (°) Female	5.6 ± 0.7

According to PREDIMED score, the majority of subjects included in this study reached a high-average adherence to the Mediterranean diet, while only one third of the subjects reported a low adherence. Results are expressed as mean ± SD or as median plus range according to variable distributions evaluated by Kolmogorov-Smirnov test. Frequencies are expressed as number and percentage. BMI, Body Mass Index; PREDIMED, PREvención con DIeta MEDiterránea, R, Resistance; Xc, Reactance; PhA, Phase Angle.

**Table 2:** Descriptive data of PREDIMED score stratified by sex, age, and BMI.

BMI	Males *n* = 461					Females *n* = 552					*p*
	PREDIMED Score					PREDIMED Score					
	*n*	Mean	SD	10th	5th	*n*	Mean	SD	10th	5th	
Normal weight											
18–28 years	25	6.9	±3.34	3.00	2.20	31	10.0	±2.51	5.00	4.50	**<0.001**
29–38 years	15	11.0	±1.79	9.00	8.70	25	12.1	±1.94	11.40	9.40	**0.041**
39–48 years	14	11.1	±2.09	8.30	7.30	31	10.9	±1.45	9.00	8.50	0.748
49–58 years	12	8.5	±2.97	4.20	3.10	11	8.8	±2.40	5.00	4.50	0.779
*p*		**<0.001**					**<0.001**				
Overweight											
18–28 years	33	6.7	±2.82	4.00	3.00	27	8.5	±2.71	5.00	4.30	**0.001**
29–38 years	30	8.8	±2.03	5.90	5.00	13	10.7	±1.43	8.40	8.00	**0.012**
39–48 years	27	9.3	±2.22	5.60	5.00	31	9.2	±2.40	7.00	6.00	0.818
49–58 years	27	7.7	±2.70	4.00	3.30	14	7.5	±3.37	4.00	3.65	0.874
*p*		**<0.001**					**0.013**				
Obesity grade I											
18–28 years	32	6.2	±3.09	2.00	1.55	34	7.2	±1.79	5.00	5.00	**0.008**
29–38 years	17	7.8	±2.46	4.20	3.00	25	9.6	±1.15	9.00	8.20	**0.001**
39–48 years	16	7.6	±2.85	4.00	2.75	27	8.3	±1.54	6.60	6.00	0.353
49–58 years	22	6.8	±2.34	3.00	3.00	22	6.0	±2.33	4.00	4.00	0.252
*p*		**0.198**					**<0.001**				
Obesity grade II											
18–28 years	27	4.2	±2.05	2.00	1.30	33	5.6	±1.67	4.00	3.60	**0.003**
29–38 years	18	8.4	±1.50	6.70	5.85	30	8.1	±1.88	5.00	5.00	0.489
39–48 years	21	6.4	±1.80	5.00	4.00	30	7.2	±1.90	4.00	4.00	**0.026**
49–58 years	21	5.0	±1.38	3.00	3.00	23	6.3	±2.18	4.00	4.00	**0.011**
*p*		**<0.001**					**<0.001**				
Obesity grade III											
18–28 years	30	3.0	±1.35	1.90	1.00	31	4.0	±0.91	3.00	2.50	**0.001**
29–38 years	21	6.7	±1.80	5.00	4.00	52	6.4	±1.96	5.00	5.00	0.412
39–48 years	33	4.4	±2.13	2.00	1.60	39	4.7	±0.76	4.00	4.00	0.641
49–58 years	20	2.7	±1.18	1.00	1.00	23	3.6	±1.03	2.00	2.00	**0.007**
*p*		**<0.001**					**<0.001**				

Across all BMI categories, females showed an overall higher adherence to Mediterranean diet compared with males up to 18–28 age years. Within each sex and BMI group, PREDIMED score increased significantly up to 29–38 years, with a subsequent decline along with increasing age. In contrast, within each sex and age group PREDIMED score tended to decrease with increasing BMI. Results are expressed as mean ± SD. *p* value in bold type denotes a significant difference (*p* < 0.05). PREDIMED, PREvención con DIeta MEDiterránea, BMI, Body Mass Index; SD, Standard Deviation.

**Table 3:** Differences of PhA in study population stratified by sex, age, and BMI.

BMI	Males *n* = 461					Females *n* = 552					*p*
	PhA					PhA					
	*n*	Mean	SD	10th	5th	*n*	Mean	SD	10th	5th	
Normal weight											
18–28 years	25	6.08	±0.43	5.60	5.52	31	5.65	±0.21	5.40	5.25	**<0.001**
29–38 years	15	6.72	±0.39	6.30	6.27	25	6.07	±0.16	5.90	5.90	**<0.001**
39–48 years	14	6.84	±0.38	6.43	6.33	31	5.74	±0.15	5.60	5.60	**<0.001**
49–58 years	12	6.10	±0.34	5.60	5.60	11	5.52	±0.06	5.50	5.50	**<0.001**
*p*		**<0.001**					**<0.001**				
Overweight											
18–28 years	33	6.58	±0.62	5.92	5.80	27	6.30	±0.22	6.00	6.00	**0.001**
29–38 years	30	7.24	±0.46	6.79	6.48	13	6.86	±0.12	6.80	6.76	**<0.001**
39–48 years	27	6.86	±0.32	6.40	6.40	31	6.47	±0.25	6.3	6.2	**<0.001**
49–58 years	27	6.41	±0.45	5.90	5.90	14	5.98	±0.33	5.63	5.53	**<0.001**
*p*		**<0.001**					**<0.001**				
Obesity grade I											
18–28 years	32	5.88	±0.73	4.90	4.90	34	5.55	±0.28	5.13	5.10	**0.021**
29–38 years	17	7.08	±0.51	6.34	6.08	25	6.40	±0.37	6.10	5.94	**<0.001**
39–48 years	16	6.72	±0.47	6.10	6.00	27	5.85	±0.29	5.46	5.40	**<0.001**
49–58 years	22	5.54	±0.45	4.82	4.70	22	5.21	±0.38	4.81	4.70	**0.012**
*p*		**<0.001**					**<0.001**				
Obesity grade II											
18–28 years	27	5.39	±0.70	4.46	4.26	33	5.10	±0.35	4.70	4.66	**0.041**
29–38 years	18	6.53	±0.30	6.10	6.08	30	6.13	±0.29	5.89	5.74	**<0.001**
39–48 years	21	5.93	±0.40	5.50	5.40	30	5.36	±0.41	4.90	4.84	**<0.001**
49–58 years	21	5.23	±0.61	4.40	4.30	23	4.55	±0.30	4.20	4.20	**<0.001**
*p*		**<0.001**					**<0.001**				
Obesity grade III											
18–28 years	30	4.96	±0.40	4.39	4.30	31	4.63	±0.31	4.20	4.15	**<0.001**
29–38 years	21	6.31	±0.27	6.00	5.97	52	5.61	±0.47	5.10	5.10	**<0.001**
39–48 years	33	5.73	±0.44	5.22	5.16	39	4.99	±0.21	4.70	4.69	**<0.001**
49–58 years	20	4.8	±0.34	4.40	4.39	23	4.50	±0.29	4.12	4.01	**<0.001**
*p*		**<0.001**					**<0.001**				

Across all age and BMI categories PhA was significantly greater in males than in females. Within each sex and BMI group, PhA tended to increase with increasing age up to 29–38 years, except for the normal weight men whose PhA continued to increase up to 39–48 years, with a subsequent decline along with increasing age. Within each age group, PhA tended to increase with increasing BMI up to a value of 25–30 kg/m^2^ in both females and males, while decreasing in higher BMI groups. Results are expressed as mean ± SD. *p* value in bold type denotes a significant difference (*p* < 0.05). PhA, Phase Angle, BMI, Body Mass Index; SD, Standard Deviation.

**Table 4:** Corresponding values of PhA according to PREDIMED score in study population divided according to sex, after adjusting for age and BMI.

PREDIMED Score	Males *n* = 461		Females *n* = 552	
	*n*	Mean ± SD	*n*	Mean ± SD
Low adherence				
1	13	4.89 ± 0.33	0	-
2	32	5.08 ± 0.63	6	4.10 ± 0.16
3	46	5.40 ± 0.73	19	4.68 ± 0.59
4	38	5.58 ± 0.64	71	4.85 ± 0.39
5	65	5.92 ± 0.77	94	5.24 ± 0.42
Average adherence				
6	35	6.14 ± 0.66	36	5.32 ± 0.43
7	31	6.28 ± 0.54	54	5.66 ± 0.50
8	43	6.53 ± 0.62	54	5.81 ± 0.52
9	67	6.45 ± 0.57	76	5.94 ± 0.47
High adherence				
10	36	6.61 ± 0.45	35	6.00 ± 0.45
11	27	6.92 ± 0.39	40	6.04 ± 0.62
12	18	6.96 ± 0.41	38	6.10 ± 0.44
13	7	6.97 ± 0.72	27	6.12 ± 0.41
14	3	6.99 ± 0.29	2	6.15 ± 0.21

In both sexes, the highest PhA values were observed among the subjects reaching the highest PREDIMED score. Results are expressed as mean ± SD. PREDIMED, PREvención con DIeta MEDiterránea, SD, Standard Deviation.

**Table 5:** Correlations between PREDIMED score and PhA in study population divided according to sex and BMI categories.

Body Weight	*n*	PhA				*n*	PhA			
		Males *n* = 461					Females *n* = 552			
		Simple Correlation		After Adjuster for Age and BMI			Simple Correlation		After Adjuster for Age and BMI	
		*r*	*p*	*r*	*p*		*r*	*p*	*r*	*p*
PREDIMED score	66	0.501	**<0.001**	0.362	**0.003**	98	0.683	**<0.001**	0.357	**<0.001**
Normal weight										
PREDIMED score	117	0.516	**<0.001**	0.617	**<0.001**	85	0.508	**0.001**	0.503	**<0.001**
Over weight										
PREDIMED score	87	0.439	**<0.001**	0.428	**<0.001**	108	0.743	**<0.001**	0.736	**<0.001**
Obesity grade I										
PREDIMED score	87	0.733	**<0.001**	0.678	**<0.001**	116	0.512	**<0.001**	0.558	**<0.001**
Obesity grade II										
PREDIMED score	104	0.680	**<0.001**	0.455	**<0.001**	145	0.862	**<0.001**	0.751	**<0.001**
Obesity grade III										

PhA exhibited a gender difference in the association with BMI, as PhA and BMI were positively associated in normal weight and overweight categories in male participants, but only in normal weight category in females. In both sexes, along with increasing BMI, a negative association was present starting from obesity III categories. This association remained significant also after adjusting for age. *p* value in bold type denotes a significant difference (*p* < 0.05). PhA, Phase Angle; BMI, Body Mass Index; PREDIMED, PREvención con DIeta MEDiterránea.

**Table 6:** Multiple Regression analysis models (stepwise method) with the PhA as dependent variables to estimate the predictive value of PREDIMED score, BMI and age in male (model 1) and female participants (model 2).

Step	Variable Inserted	*p* Value	β	*t*	*R* ^2^	95% CI
Model 1 Males						
1	PREDIMED score	**<0.001**	0.667	19.18	0.445	0.160–0.197
2	PREDIMED score BMI	**<0.001**	0.476	11.75	0.445	0.106–0.149
		**<0.001**	−0.324	−7.99	0.514	−0.041–−0.025
3	PREDIMED score BMI Age	**<0.001**	0.486	12.03	0.444	0.109–0.151
		**<0.001**	−0.315	−7.79	0.511	−0.040–−0.024
		**0.009**	−0.086	−2.63	0.521	−0.011–−0.002
Model 2 Females						
1	PREDIMED score	**<0.001**	0.688	22.21	0.473	0.145–0.173
2	PREDIMED score BMI	**<0.001**	0.545	13.08	0.473	0.107–0.145
		**<0.001**	−0.208	−5.00	0.496	−0.025–−0.011
3	PREDIMED score BMI Age	**<0.001**	0.533	12.09	0.473	0.105–0.142
		**<0.001**	−0.211	−5.12	0.496	−0.025–−0.011
		**<0.001**	−0.109	0.508	0.508	−0.010–−0.003

In both model 1 (male participants) and 2 (female participants), PREDIMED score entered at the first step and appeared to be the factor exerting the most powerful influence on PhA, while BMI entered at the second step, followed by age. *p* value in bold type denotes a significant difference (*p* < 0.05). BMI, Body Mass Index; PhA, Phase Angle; CI, Confidence Interval.

## Supplementary Materials

The following are available online at www.mdpi.com/2072-6643/9/2/151/s1.

## Abbreviations

The following abbreviations are used in this manuscript:

PhA	Phase Angle
BMI	Body Mass Index
PREDIMED	PREvención con DIeta MEDiterránea
