# Supplementary material for: Mediterranean Diet and Phase Angle in a Sample of Adult Population: Results of a Pilot Study
Source: Nutrients. 2017 Feb 17;9(2):151. doi: 10.3390/nu9020151 (PMC5331582; doi:10.3390/nu9020151)
Supplement: Supplementary file 1 [file nutrients-09-00151-s001.docx]

**Supplementary Table 1 (Part 1):** **Characteristics of study participants by tertile of phase angle**

| **Parameters** | **Males** | | | |
| --- | --- | --- | --- | --- |
|  | **PhA≤5.5**  n=123; | **5.6≤PhA≤6.2**  n=119; | **PhA≥6.3**  n=219; | ***p* value** |
| Age (years) | 43.0 (18.0 – 58.0) | 40.0 (18.0 – 57.0) | 34.0 (18.0 – 56.0) | ***p*<0.001** |
| ***Anthropometric measures*** |  |  |  |  |
| BMI (kg/m^2^) | 43.0 (20.9 – 57.9) | 33.0 (21.3 – 45.3) | 28.2 (21.1 – 47.2) | ***p*<0.001** |
| *Normal weight*  *n (%)* | 2; 1.6% | 21; 17.6% | 43; 19.6% | χ^2^=22.27,  ***p*<0.001** |
| *Overweight*  *n (%)* | 2; 1.6% | 20; 16.9% | 94; 42.9% | χ^2^=77.28,  ***p*<0.001** |
| *Obesity grade I*  *n (%)* | 20; 16.3% | 28; 23.5% | 39; 17.8% | χ^2^=2.40,  *p*=0.302 |
| *Obesity grade II*  *n (%)* | 35; 28.5% | 29; 24.4% | 23; 10.5% | χ^2^=19.75,  ***p*=0.001** |
| *Obesity grade III*  *n (%)* | 64; 52.0% | 21; 17.6% | 20; 9.2% | χ^2^=84.81,  ***p*<0.001** |
| ***Adherence to the MD*** |  |  |  |  |
| PREDIMED score | 3.4±1.7 | 6.6±2.4 | 8.5±2.6 | ***p*<0.001** |
| *Low adherence*  *n (%)* | 110; 89.4% | 44; 37.0% | 38; 17.4% | χ^2^=169.82,  ***p*<0.001** |
| *Average adherence*  *n (%)* | 13; 10.6% | 65; 54.6% | 100; 45.6% | χ^2^=58.27,  ***p*<0.001** |
| *High adherence*  *n (%)* | 0; 0% | 10; 8.4% | 81; 37.0% | χ^2^=82.91,  ***p*<0.001** |

| **Parameters** | **Females** | | | |
| --- | --- | --- | --- | --- |
|  | **PhA≤5.1**  n=158; 28.6% | **5.2≤PhA≤5.6**  n=119; 21.6% | **PhA≥5.7**  n=275; 49.8% | ***p* value** |
| Age (years) | 42.0 (18.0 – 57.0) | 38.0 (18.0 – 57.0) | 34.0 (18.0 – 56.0) | ***p*<0.001** |
| ***Anthropometric measures*** |  |  |  |  |
| BMI (kg/m^2^) | 43.3 (19.6 – 49.8) | 34.0 (19.5 – 45.2) | 29.2 (20.4 – 42.6) | ***p*<0.001** |
| *Normal weight*  *n (%)* | 1; 0.6% | 30; 25.2% | 67; 24.4% | χ^2^=44.48,  ***p*<0.001** |
| *Overweight*  *n (%)* | 0; 0% | 3; 2.5% | 82; 29.8% | χ^2^=34.98,  ***p*<0.001** |
| *Obesity grade I*  *n (%)* | 13; 8.2% | 30; 25.2% | 65; 23.6% | χ^2^=18.21,  ***p*=0.001** |
| *Obesity grade II*  *n (%)* | 52; 32.9% | 24; 20.2% | 40; 14.5% | χ^2^=20.46,  ***p*<0.001** |
| *Obesity grade III*  *n (%)* | 92; 58.3% | 32; 26.9% | 21; 7.6% | χ^2^=132.6,  ***p*<0.001** |
| ***Adherence to the MD*** |  |  |  |  |
| PREDIMED score | 4.7±1.5 | 6.9±2.2 | 9.4±2.3 | ***p*<0.001** |
| *Low adherence*  *n (%)* | 129; 81.6% | 43; 36.1% | 19; 6.9% | χ^2^=247.8,  ***p*<0.001** |
| *Average adherence*  *n (%)* | 27; 17.1% | 6; 52.1% | 130; 47.3% | χ^2^=87.65,  ***p*<0.001** |
| *High adherence*  *n (%)* | 2; 1.3% | 14; 11.8% | 126; 45.8% | χ^2^=119.7,  ***p*<0.001** |

**Supplementary Table 1 (Part 2):** **Characteristics of study participants by** **tertile of phase angle**

**Footnotes to Supplementary Table 2 part 1 and 2:**

Results are expressed as mean±SD or as median plus range according to variable distributions evaluated by Kolmogorov-Smirnov test. The frequencies are expressed as number and percentage. Differences between groups were analyzed by ANOVA or Kruskal-Wallis test, when appropriate. The chi square (χ^2^) test was used to determine significance differences in frequency distributions of BMI and PREDIMED classes. A *p* value in bold type denotes a significant difference (*p*<0.05).

***PhA,*** Phase Angle; ***BMI,*** Body Mass Index; ***MD,*** Mediterranean Diet; ***PREDIMED,*** PREvención con DIeta MEDiterránea.

**Supplementary Table 2: Correlations between PREDIMED score and BMI in study population divided according to sex and BMI categories.**

| Body Weight | **PREDIMED score**  **Males**  *n=461* | | | | | **PREDIMED score**  **Females**  *n=552* | | | | |
| --- | --- | --- | --- | --- | --- | --- | --- | --- | --- | --- |
|  | Simple  correlation | | | After adjuster  for age | | Simple  correlation | | | After adjuster  for age | |
|  | n | r | *p* | r | *p* | n | r | *p* | r | *p* |
| **BMI**  *Normal*  *weight* | 66 | 0.450 | **<0.001** | 0.386 | **<0.001** | 98 | 0.628 | **<0.001** | 0.628 | **<0.001** |
| **BMI**  *Over*  *weight* | 117 | -0.242 | **0.009** | -0.235 | **0.011** | 85 | -0.222 | **0.042** | -0.223 | **0.041** |
| **BMI**  *Obesity*  *grade I* | 87 | -0.458 | **<0.001** | -0.446 | **<0.001** | 108 | -0.278 | **0.004** | -0.235 | **0.015** |
| **BMI**  *Obesity*  *grade II* | 87 | -0.451 | **<0.001** | -0.443 | **<0.001** | 116 | -0.443 | **<0.001** | -0.450 | **<0.001** |
| **BMI**  Obesity  grade III | 104 | -0.548 | **<0.001** | -0.563 | **<0.001** | 145 | -0.623 | **<0.001** | -0.634 | **<0.001** |

**Footnotes to Supplementary Table 2**:

A significant positive correlation was observed between PREDIMED score and BMI in normal weight in both sexes in unadjusted data. Along with increasing BMI, this correlation became negative and remained significant also after adjusting for age, in each sex and BMI categories. A *p* value in bold type denotes a significant difference (*p*<0.05).

***PREDIMED,*** PREvención con DIeta MEDiterránea, ***BMI,*** Body Mass Index.

**Supplementary Table 3: Correlations between PhA and BMI in study population divided according to sex and BMI categories.**

| Body Weight | n | **PhA**  **Males**  *n=461* | | | | n | **PhA**  **Females**  *n=552* | | | |
| --- | --- | --- | --- | --- | --- | --- | --- | --- | --- | --- |
|  |  | Simple  correlation | | After adjuster  for age | |  | Simple  correlation | | After adjuster  for age | |
|  |  | r | *p* | r | *p* |  | r | *p* | r | *p* |
| **BMI**  *Normal*  *weight* | 66 | 0.476 | **<0.001** | 0.445 | **0.008** | 98 | 0.870 | **<0.001** | 0.870 | **<0.001** |
| **BMI**  *Over*  *weight* | 117 | 0.216 | **0.019** | 0.210 | **<0.001** | 85 | -0.048 | 0.484 | -0.051 | 0.646 |
| **BMI**  *Obesity*  *grade I* | 87 | -0.165 | 0.126 | -0.185 | 0.682 | 108 | -0.140 | 0.287 | -0.077 | 0.431 |
| **BMI**  *Obesity*  *grade II* | 87 | -0.457 | **<0.001** | -0.507 | 0.053 | 116 | -0.192 | **0.626** | -0.182 | 0.052 |
| **BMI**  *Obesity*  *grade III* | 104 | -0.814 | **<0.001** | -0.823 | **<0.001** | 145 | -0.759 | **<0.001** | -0.773 | **<0.001** |

**Footnotes to Supplementary Table 3**:

A significant positive correlation was observed between PREDIMED score and PhA along BMI categories in both sexes in unadjusted data. Of interest, PhA remained positively correlated with PREDIMED score independently of age and BMI. A *p* value in bold type denotes a significant difference (*p*<0.05).

***PhA,*** Phase Angle; ***BMI,*** Body Mass Index.
